# Contrast Enhanced Ultrasound of the Kidneys: What Is It Capable of?

**DOI:** 10.1155/2013/595873

**Published:** 2013-12-24

**Authors:** Demosthenes D. Cokkinos, Eleni G. Antypa, Maria Skilakaki, Despoina Kriketou, Ekaterini Tavernaraki, Ploutarchos N. Piperopoulos

**Affiliations:** Radiology Department, Evangelismos Hospital, 45-47 Ypsilantou Street, 10676 Athens, Greece

## Abstract

One of the many imaging uses of contrast enhanced ultrasound (CEUS) is studying a wide variety of kidney pathology, due to its ability to detect microvascular blood flow in real time without affecting renal function. CEUS enables dynamic assessment and quantification of microvascularisation up to capillary perfusion. The objective of this paper is to briefly refresh basic knowledge of ultrasound (US) contrast agents' physical properties, to study technical details of CEUS scanning in the kidneys, and to review the commonest renal indications for CEUS, with imaging examples in comparison to baseline unenhanced US and computed tomography when performed. Safety matters and limitations of CEUS of the kidneys are also discussed.

## 1. Introduction

Contrast enhanced ultrasound (CEUS) is an imaging technique that has gained in the last decade high acceptance among radiologists. It allows real-time evaluation of microvasculature which Colour Doppler ultrasound (US) cannot detect. CEUS can be performed for a wide variety of indications in practically all parts of the human body. It is of particular usefulness for answering many clinical questions in the kidneys, including detection and characterisation of lesions, based on differences between lesion and organ perfusion, differentiation between solid renal masses and pseudotumours, as well as between cystic and solid lesions. It can also be performed for characterising complex cystic renal masses and grading them with the Bosniak system, imaging renal ischaemia, infections, and trauma, as well as facilitating vascular imaging for renal artery stenosis. Finally, CEUS can be performed for the assessment of percutaneous ablation therapy for kidney tumours. This technique offers many advantages in comparison to other imaging modalities, a very important one being that US contrast agents do not affect renal function. It can be easily used in routine clinical practice, improving detection and characterisation of many entities and reducing the number of additional imaging examinations.

## 2. What Are US Contrast Agents?

These agents are composed of gas microbubbles enclosed in a protein, lipid, or polymer shell [1]. This composition combination allows the agent to be able to last for a certain period of time (in practice up to 5–7 min) inside the blood vessel. The microbubble diameter ranges from 1 to 10 *μ*m, which is in general the size of a red blood cell. As a consequence, these drugs show no extravascular passage and are regarded as pure blood pool agents [2].

When these agents are exposed to a US wave, bubbles contract and expand in almost double their diameter at a resonance frequency which, by coincidence, is close to the frequencies used for diagnostic US imaging. During this oscillation, they send back to the US machine transducer an amount of energy higher than that of passive reflectors like computed tomography (CT) or magnetic resonance (MR) contrast agents. Their expansion during rarefaction is higher than the following contraction during pressure. This asymmetric oscillation produces a returning signal containing harmonics [3], which are US signals with frequency peaks at multiple of the original insonation frequencies sent by the machine probe [1].

After circulating for several minutes inside the blood vessel lumen, microbubbles dissolve: the internal gas is exhaled by the lungs and the coating shell is metabolised, basically in the liver [4]. The kidneys play no part in their excretion and microbubbles do not accumulate in the pelvicalyceal system, which therefore does not enhance as in intravenous urography or contrast enhanced CT (CECT). In addition, because of this metabolic pathway, renal insufficiency poses no contraindication for the use of these agents.

US contrast agents have the ability to detect microvasculature in vessels too small and with very low velocity that may be overlooked by Colour and Power Doppler. In fact, Doppler US can image blood vessels as small as 100 *μ*m, while CEUS spatial resolution can show vessels as small as 40 *μ*m [5]. They can be imaged with higher temporal resolution in real time continuously in comparison to CT and MR agents, where only still images in specific time slots can be observed. Enhancement patterns are grossly similar to those of CT/MR contrast uptake [4, 6] but not identical, since the latter are cleared from the blood pool into the extracellular space.

## 3. Physics and US Equipment Specifications

In order to perform CEUS examinations, a US machine should be equipped with imaging techniques capable of detecting contrast agents. The most widely available of these techniques is based on the principle of phase inversion: two different US pulses, 180° out of phase between them, are sent consecutively. Echoes returning to the transducer are added up by the US machine [7]. As a result, linear echoes reflected by the different body tissues nullify each other, whereas nonlinear echoes returned by the microbubbles generate a strong signal. In this way, signals from tissues are cancelled almost completely, but the contrast agent is observed with a very strong signal. An adequate period of agent imaging is guaranteed if the machine's mechanical index (MI) is kept at a low level, allowing best agent detection and minimal bubble destruction.

Most US machines equipped with CEUS imaging technique have a split-screen view, where contrast enhancement is presented side by side to nonenhanced, gray scale image (Dual View Mode). This facilitates the examiner's orientation in the area of interest, while at the same time observing the enhancement pattern. In our department we administer one of the more commonly used agents, SonoVue, which consists of stabilised aqueous suspension of sulfur hexafluoride microbubbles with a phospholipid shell. The dose for kidney imaging ranges between 1 and 2.4 mL, depending on the type of machine used and the body habitus of the patient scanned.

## 4. Safety

US contrast agents are very safe, with a very low rate of anaphylactoid reactions (1 : 7000 patients, 0.014%) [8–10], which is lower than the comparable rate of CT agents (0.035–0.095%) [8, 11, 12]. However, as in all drugs, precautions should be kept in mind for certain patient groups. These mainly include cases of recent cardiopulmonary pathology (myocardial infarction, ongoing angina, recent coronary artery intervention or electrocardiogram changes, recurrent episodes of angina in the last week, heart failure, serious lung disease, dyspnoea, etc.) [13]. In the United States, due to a warning given in the past by the Food and Drug Administration (FDA), which was recently modified, these agents are not used clinically except in echocardiography. However, throughout the world, contrast agents are being administered safely. The FDA warning has been considered to “ignore the proven efficacy of US contrast agents, the previously established safety of these compounds, the potential risks of alternative procedures, and the effect of pseudocomplication” [13].

A very important advantage of these agents is that, since not excreted by the kidneys, they do not affect renal function. Therefore, they can be safely administered to patients with renal insufficiency, while blood tests are not needed prior to their injection in order to assess kidney function. This is very useful in cases of CT or MR studies that cannot be carried out with contrast administration, with CEUS being the only modality that offers dynamic assessment of perfusion of the organ in question.

## 5. Renal CEUS

After contrast injection, enhancement can be detected in real time for up to 5–7 minutes in the liver or spleen. However, kidneys enhance for a shorter period of time. The arterial pedicle and main branches pick up the agent first. After a few seconds, the cortex enhances, followed by medullary perfusion. The outer medulla fills in earlier, while the pyramids fill in gradually later [14]. Satisfactory uptake usually lasts for 2 min in the kidneys, and subsequently contrast concentration in circulation decreases and enhancement fades. In chronic renal disease kidneys, enhancement is poorer and shorter, fading earlier [15].

## 6. Indications for Renal CEUS

As in most medical fields, when a new technique emerges, it is initially used in a wide variety of indications. Following the publication of clinical studies results, more appropriate indications for correct usage are identified. The same has happened in the last years with CEUS. The 2011 updated European Federation of Societies for Ultrasound in Medicine and Biology (EFSUMB) Guidelines and Recommendations on the Clinical Practice of CEUS [15] have identified the current indications for the administration of US contrast agents for studying different parts of the body, including the kidneys. According to these guidelines, CEUS should be used to answer specific clinical questions in the kidneys. In our practice, we have accumulated experience on most of the fields outlined by the EFSUMB Guidelines, which will be reviewed in detail.

### 6.1. Differential Diagnosis between Solid Renal Masses and Pseudotumours

**Figure 1:**
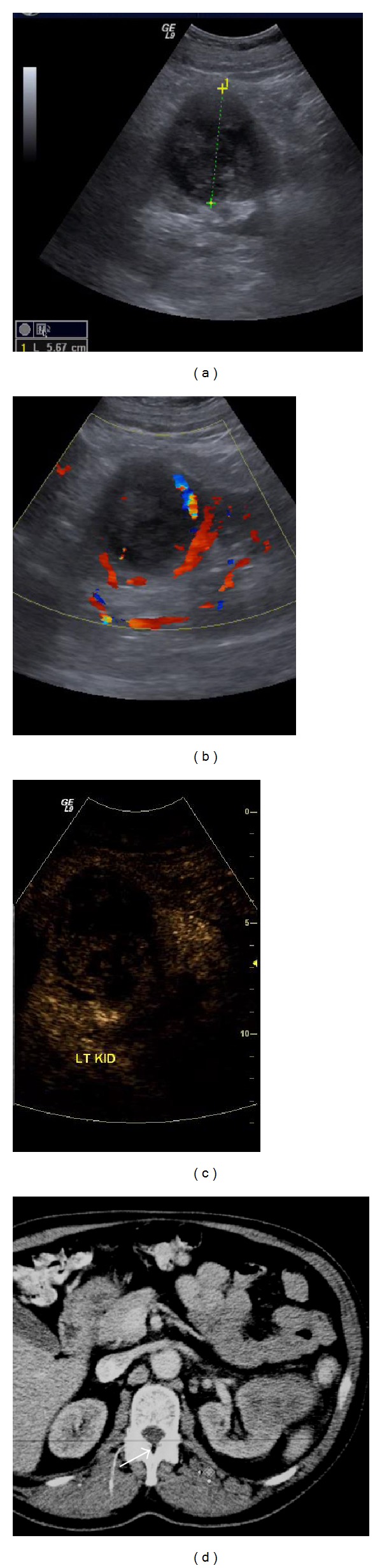
Renal cell carcinoma: a large mixed echogenicity lesion is seen in the middle of the left kidney on B-mode US (a). Colour Doppler (b) reveals some peripheral blood flow. On CEUS (c) there is uptake inside the lesion, but altogether different enhancement than the rest of the kidney. Contrast enhanced CT (d) confirms the mass. Histology after surgery diagnosed a renal cell carcinoma.

**Figure 2:**
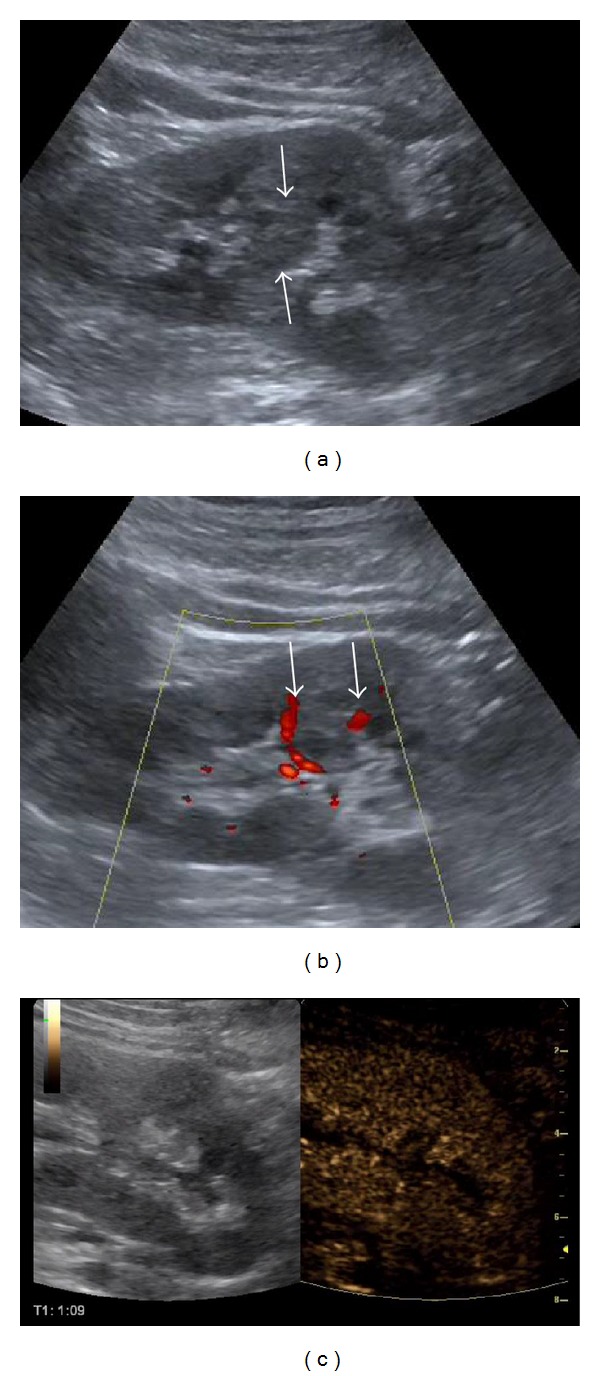
Kidney pseudotumour: a solid isoechoic area is noted in the middle of the left kidney on B-mode US (arrows in (a)), seeming to displace blood vessels on Colour Doppler (arrows in (b)). After SonoVue injection (c), this area enhances in the same way as the rest of the renal parenchyma, suggestive of a pseudotumour of no clinical significance.

In general, regardless of echogenicity, the vascularity of renal tumours is different from normal parenchyma, at least in one vascular phase [15], with any area enhancing differently considered suspicious (Figure 1). This is achieved with perfusion analysis and assessment of tissue macro- and microvascularisation and is very helpful when differentiating masses from normal variants, like a prominent Bertin septum [16]. Pseudotumours enhance parallel to the adjacent kidney parenchyma in all phases (Figure 2) [14].

However, solid tumours do not show specific perfusion patterns after injection of US contrast agents. There have been published studies concluding that CEUS can differentiate between malignant and benign solid renal masses [17]. In particular, it has been found that all malignant lesions are hypoechoic in comparison to normal renal parenchyma irrespective of the pattern of uptake in the arterial phase. In addition, several other criteria have been proposed for differentiating benign from malignant pathology [17, 18]. In practice, this discrimination is usually difficult or impossible. Therefore, CEUS is currently not used for differentiating between benign and malignant kidney lesions [15], contrary to liver studies where specific characterisation is very often possible.

**Figure 3:**
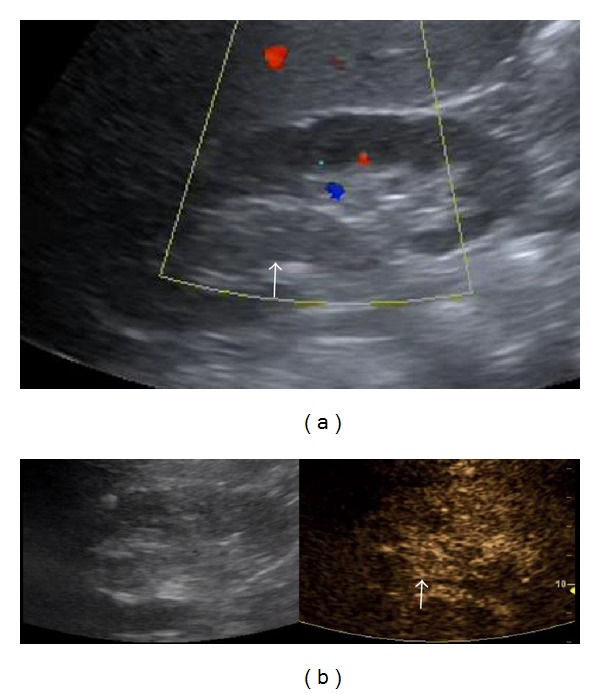
Transitional cell carcinoma: echogenic content is located in the upper part of the dilated pelvicalyceal system. It does not show blood flow on Colour Doppler US (arrow in (a)). However, it enhances on CEUS (arrow in (b)), due to its neoplastic nature.

Nevertheless, it is feasible to identify malignant renal vein invasion using CEUS, with accuracy similar to CECT [19]. Enhancing thrombus is secondary to neoplastic invasion, while bland thrombus does not show contrast uptake [15]. Similarly, enhancing echogenic material in the collecting system can be differentiated between neoplastic tissue and infectious matter (Figure 3).

### 6.2. Differentiation between Cystic and Solid Lesions of the Kidneys

**Figure 4:**
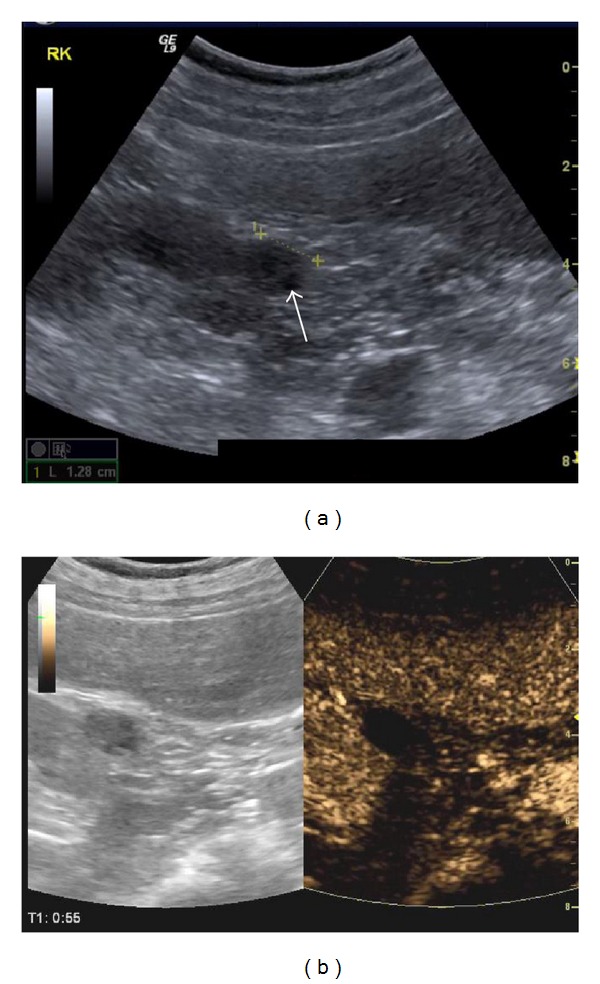
Haemorrhagic cyst: a cystic structure with some echogenic content is seen in the right kidney on B-mode US (arrow in (a)). On CEUS (b) this content shows no enhancement. This finding is not suggestive of a solid lesion but consistent with a haemorrhagic cyst.

CEUS is very helpful for evaluating atypical cysts or cyst-like lesions with echogenic content since it is more sensitive than CECT for detecting perfusion in hypovascularised lesions [20]. By detecting enhancing vessels in perfused viable tissue, contrary to nonenhancing debris, CEUS can be used in cases where differentiation between solid hypovascular tumours (which enhance, even minimally) and atypical cystic masses (where debris does not show any enhancement whatsoever) remains unanswered by CT or Colour Doppler US [15]. Thus, the cystic or solid nature of a renal lesion (Figure 4) can be based in 100% of cases on the presence of enhancement after injection [17]. Moreover, the diagnosis of cystic renal cell carcinoma using CEUS has been found to be superior to CT and MR that are occasionally indeterminate due to volume averaging [8, 21, 22].

### 6.3. Characterisation of Complex Cystic Renal Masses

Simple renal cysts detected on unenhanced US do not warrant any further imaging assessment or surgical procedure [23], but complex cystic masses with echogenic content, internal septations, thick walls, mural nodules, and calcifications may vary in malignant potential [24]. The main question that has to be answered is differentiating between complex cystic renal masses that require surgery and those that do not [25]. According to Israel and Bosniak [26, 27], baseline US is not enough to differentiate between surgical and nonsurgical complex cystic renal masses and CECT or MR is needed [24, 28]. The Bosniak classification was introduced in the 1980s [29, 30] to categorise renal cysts according to their CT features. Contrast enhancement as studied using this system, although not absolutely specific, is a crucial criterion to decide between surgical treatment and followup [31–33]. The Bosniak system is accurate for predicting malignancy [29, 30], with very high diagnostic accuracy for depicting nodular or septal enhancement [34]. Application of the Bosniak criteria on MR results in upgrading of the lesions, septations and walls being better evaluated in number, thickness, and enhancement [35].

**Figure 5:**
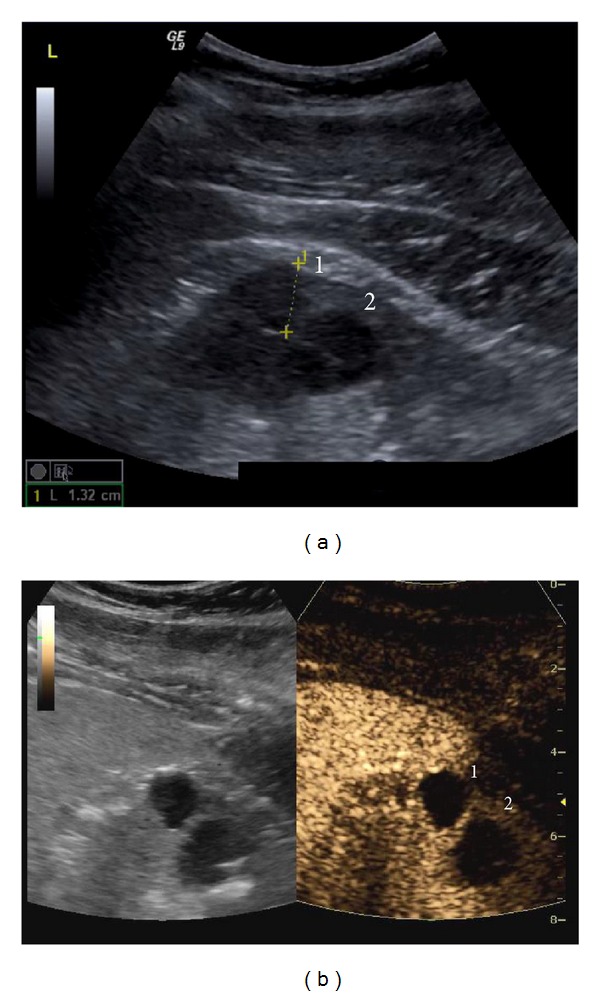
Bosniak I and II cysts: two cysts are noted on B-mode US (a). Cyst 1 shows no septa and is classified as Bosniak I. Cyst II shows a thin septum with only minimal enhancement on CEUS (b) and is classified as Bosniak II.

The Bosniak classification can also be applied to CEUS and a relevant scheme for cystic renal lesions using this modality as the reference technique was proposed [36, 37]. CEUS has shown equal or even superior diagnostic accuracy compared to CT to classify cysts using the Bosniak system [23] while even complete concordance [38] has been observed between CEUS and CECT in differentiating surgical and nonsurgical lesions in this way. CEUS has also improved characterisation of complex renal cysts that were indeterminate on CT. This may be attributed to a discrepancy between CT and CEUS in depicting septal vascularity, possibly due to the high sensitivity of the latter in detecting microbubbles in the peripheral wall or intracystic septa of the lesion, as well as showing solid enhancing components not imaged adequately by CT [18, 20, 39–41]. Thus, CEUS has been suggested to be used to evaluate every renal mass with a complex cystic appearance on baseline US (Figure 5). CT can be used for staging complex cystic renal masses with a malignant enhancement pattern on CEUS [23]. CEUS should also be considered an alternative to CT [20] for complex cysts followup to reduce radiation dose [38, 42]. No enhancement whatsoever on CEUS implies no further workup [23]. Enhancing peripheral walls, thick intracystic septa, and mural nodules after microbubble injection should be considered malignant. Minimal septal enhancement may be seen in benign cystic renal lesions. Inflammatory or haemorrhagic cysts show only peripheral wall uptake and are therefore unlikely to be misdiagnosed, as there are no intracystic septations.

**Figure 6:**
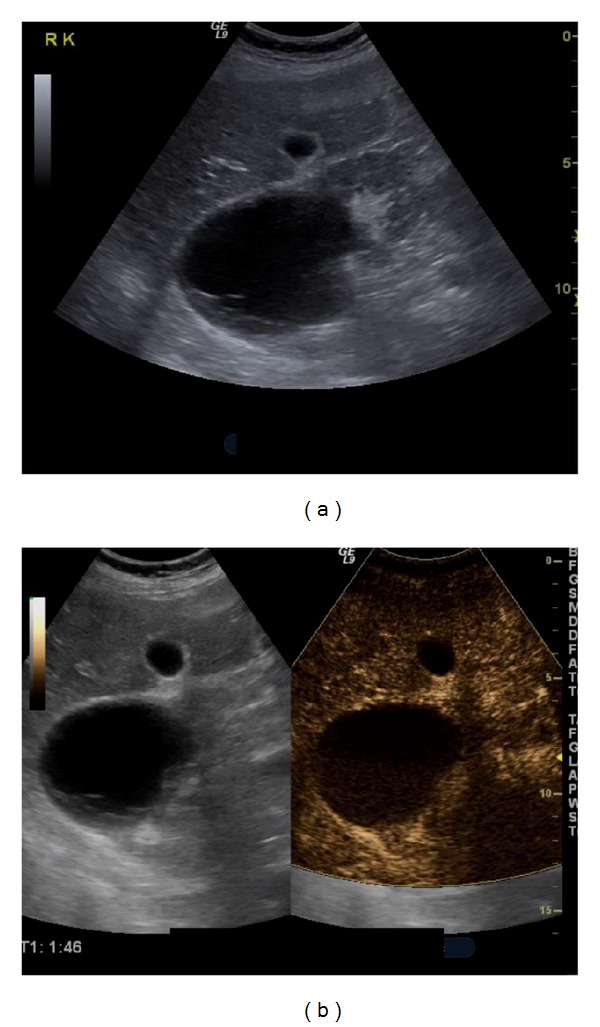
Bosniak II cyst: an anechoic cyst is seen on B-mode US (a). Some peripheral septa are present. After contrast injection (b), the septa do not show any enhancement. This classifies the cyst as Bosniak II.

**Figure 7:**
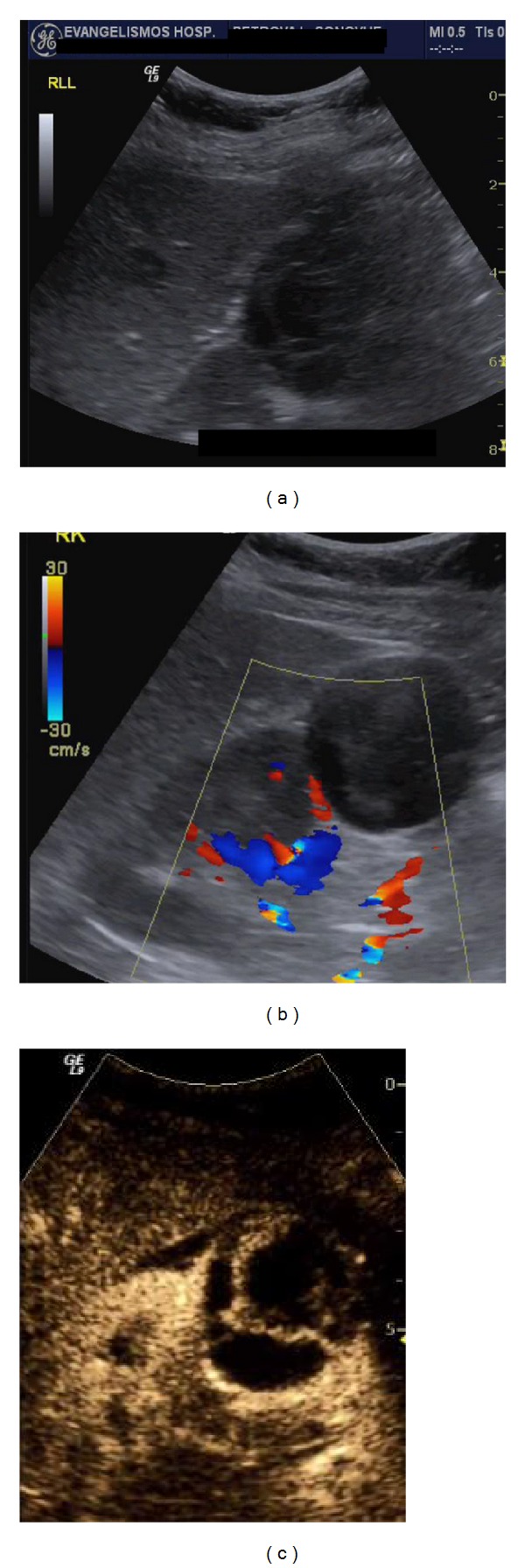
Bosniak III cyst: a mixed echogenicity cortical lesion is noted in the right kidney on B-mode US (a). Colour Doppler (b) does not reveal increased vascularity inside the lesion. However, CEUS (c) shows rich enhancement in the cyst's septa. This finding classifies the lesion as a Bosniak III renal cyst.

**Figure 8:**
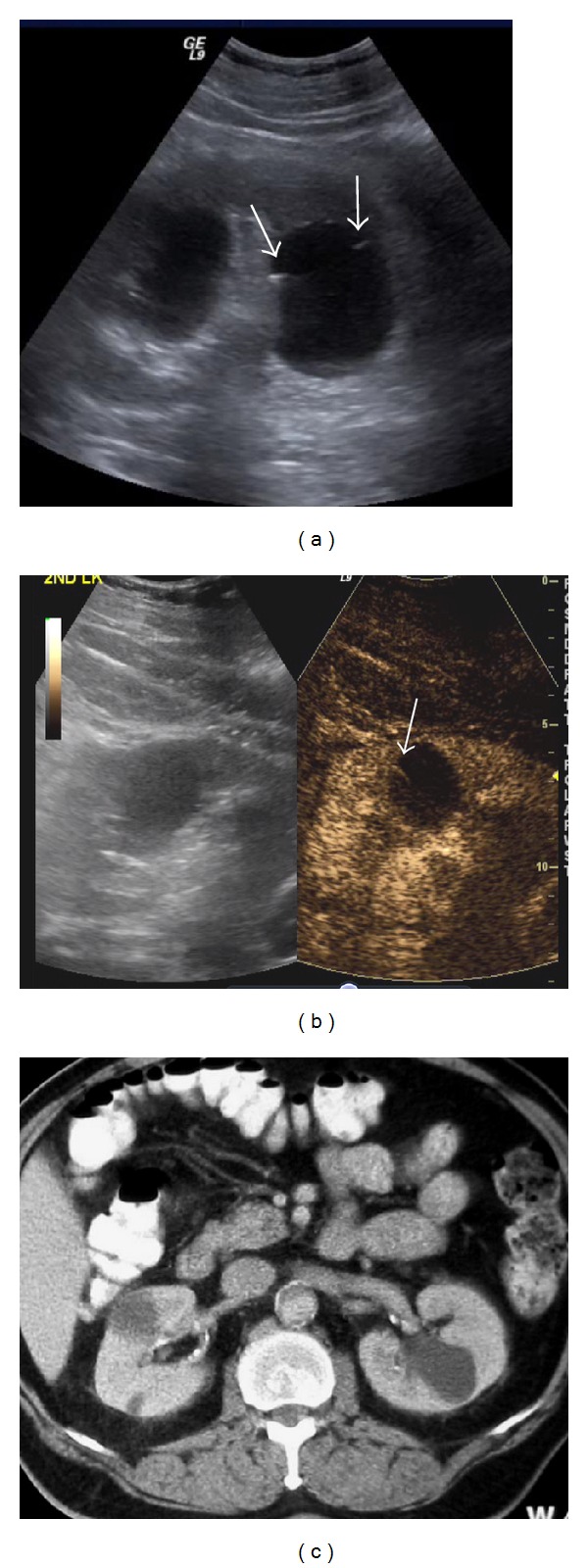
Bosniak IIF cyst: two cysts are present in the left kidney on B-mode US (a). The larger cyst shows small marginal septa (arrows). On CEUS the septa show definitive enhancement (arrow in (b)). This classifies the cyst as IIF. This enhancement is not evident on contrast enhanced CT (c).

The main difficulty when using the Bosniak classification system is differentiating between category II (Figure 6) and III (Figure 7) lesions. This is important, as deciding for intervention or not is based on this differentiation. Category IIF (Figure 8) can help in detecting those category II lesions that may eventually follow a malignant course and reduce overtreatment of lesions initially characterised as category III [43]. This overlap in malignant and nonmalignant looking cystic lesions is common, as about 10% of all renal cell carcinomas appear as complex cystic lesions [38]. On the other hand, benign renal cysts may appear complex due to haemorrhage, infection, inflammation, or ischaemia [31, 44].

Limitations of both CT and CEUS include interreader variation in distinguishing between category II, IIF, and III lesions [45]. CT also has difficulty in revealing thin intracystic septations due to volume averaging. CEUS (along with baseline US) limitations include deep location of the lesion in question, bowel gas interposition, and presence of diffuse mural calcification obscuring penetration of sonographic beam.

Altogether, CEUS can replace CT for evaluation and followup of complex renal cysts [38], especially in patients with renal insufficiency and other factors refraining them from being imaged with contrast enhanced CT or MR. It may also visualise the enhancement of some renal cystic masses better than CT, resulting in upgrading Bosniak classification and affecting their treatment plan [21]. However, CT is still the modality of reference for staging patients with malignant renal cystic lesions [15].

### 6.4. Renal Ischaemia

**Figure 9:**
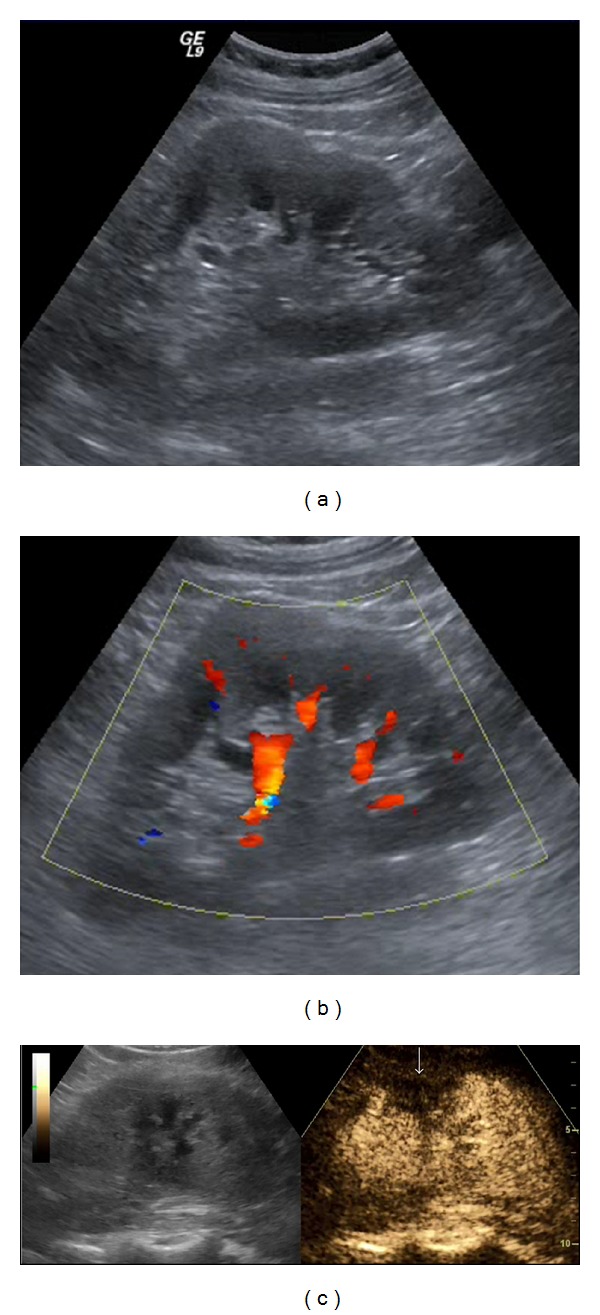
Kidney infarct: B-mode (a) and Colour Doppler (b) US detect no abnormality in the left kidney. On CEUS however (c) a triangular peripheral enhancement defect is evident (arrow).

Many studies in animals and humans have concluded that CEUS has very good diagnostic performance in the detection of kidney parenchymal ischaemia, comparable to that of CECT [15, 46]. In comparison to Colour Doppler US, CEUS is superior, detecting smaller blood vessels with slower flow, and is considered a recommended imaging technique in patients with suspected infarction. As in other organs, infarcts appear as triangular or wedge-shaped areas with no contrast uptake, while the rest of the parenchyma enhances normally [16] (Figure 9). Due to its excellent spatial resolution, CEUS allows differentiation of infarction from cortical necrosis, the latter appearing as a nonenhancing cortical area with preservation of hilar vascularity [14, 47]. In addition, CEUS can differentiate infarcts from parenchymal areas with diminished perfusion. Although both appear as areas with no flow on Doppler ultrasound, only infarcts show complete lack of contrast uptake after injection [15].

### 6.5. Renal Infections

**Figure 10:**
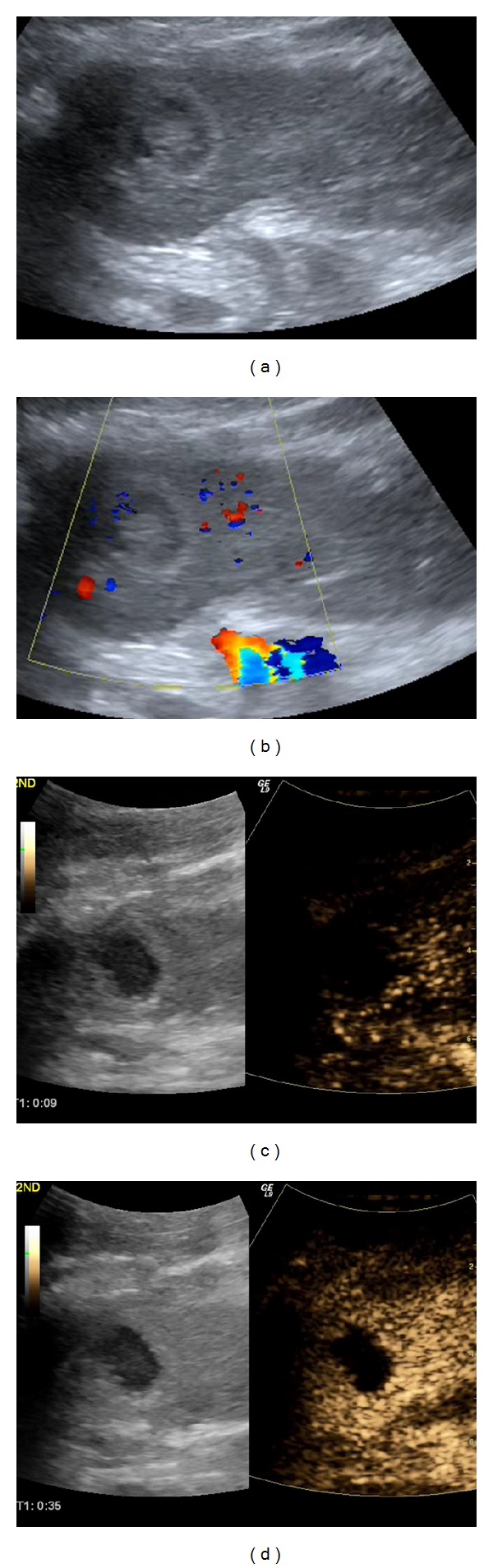
Renal abscess: B-mode (a) and Colour Doppler (b) US detect a round mixed echogenicity lesion in the upper part of the left kidney. After contrast injection, early enhancement is seen in the periphery of the lesion (c) with no internal uptake ((c), (d)).

**Figure 11:**
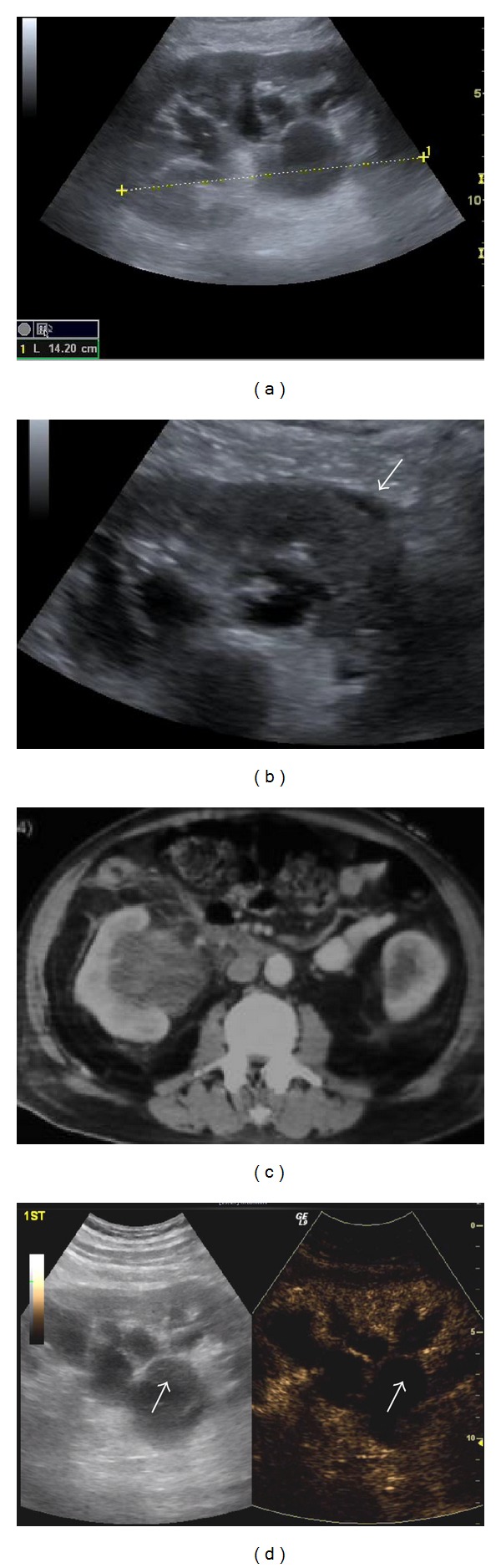
Pyelonephritis: B-mode US images an enlarged right kidney (a) with a dilated pelvicalyceal system, containing echogenic material. A small perinephric collection is also seen (arrow in (b)). CT (c) confirms these findings. On CEUS (d) the echogenic material in the collecting system (arrows) does not enhance, suggestive of a purulent, non neoplastic nature. Note the difference from transitional cell carcinoma enhancement (Figure 3).

According to the Guidelines of the European Urology Association, acute uncomplicated pyelonephritis diagnosis is established on clinical history, physical examination, and laboratory findings, with no imaging required; B-mode US may be needed only to rule out the presence of calculi and obstruction of the urinary tract [15]. Further imaging examinations are warranted if the patient is still febrile after 72 hours of treatment. However, these recommendations have a low evidence level, with no directly applicable clinical studies. Therefore CEUS and additional imaging in cases with uncomplicated pyelonephritis are debatable, with no definitive indications. In kidneys with focal pyelonephritis, areas of reduced enhancement due to oedema may be detected after contrast injection. If an abscess evolves, this appears as a nonenhancing area, with only peripheral uptake (Figure 10). CEUS can be used not only to confirm abscess detection, but also for patient followup [14]. Echogenic puss in the pelvicalyceal system or urinary bladder shows no uptake, thus being differentiated from neoplastic tissues (Figure 11).

### 6.6. Renal Trauma

Practically all trauma patients are subjected on an emergency basis to conventional unenhanced FAST (Focused Assessment with Sonography in Trauma) US. This is currently the primary imaging screening examination [48–50]. However although FAST may be excellent for detecting free abdominal, pleural, and pericardial fluids [51], its sensitivity is very low for imaging traumatic lesions of abdominal solid organs (liver, kidneys, and spleen) [52], which may be isoechoic to the surrounding parenchyma. In addition, in up to a third of cases, solid organ injuries may be present without haemoperitoneum [53, 54]. As a consequence, these injuries may be missed on conventional US. Additional limitations of US (inability for deep breath, poor imaging of gut perforation, or pancreatic trauma) make CECT the reference examination and modality of choice for high-energy multitrauma, since it offers thin organ segments in large body sections very fast, with high resolution [55]. It is exactly these advantages of CT that have led to an increase of CT scans, with long waiting on busy days. In addition, CT disadvantages include need of patient transfer from emergency to CT room, high ionising radiation amount (especially if pre- and postcontrast administration images are acquired), in an often young age group, high cost, use of iodinated contrast agents with potential adverse effects on renal function or anaphylactic reactions and imaging artefacts due to patient's inability to raise arms, and placed iatrogenic tubes, lines, and catheters [56].

**Figure 12:**
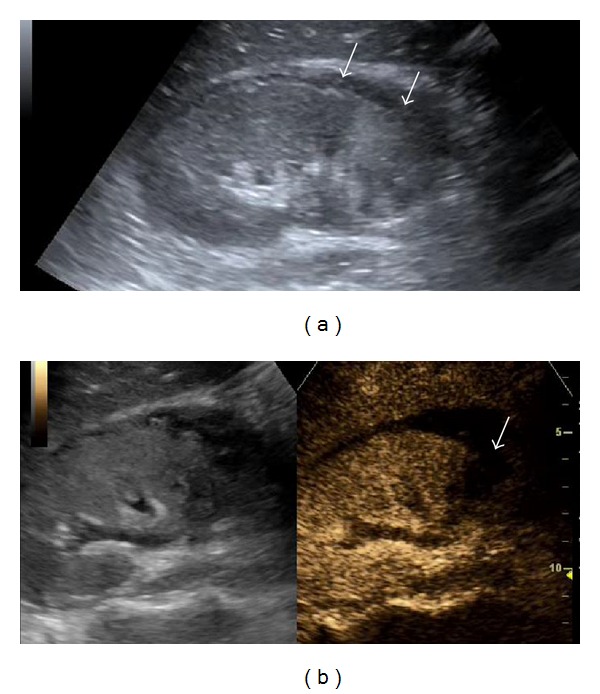
Renal trauma: B-mode US detects areas of different echogenicities in the lower moiety of the kidney (arrows in (a)). After contrast injection (b), a filling defect is seen due to kidney laceration (arrow).

A result of CT overuse is that emergency CT scans for trauma are often negative, resulting in squandering of radiation, time, and money. This is a common scenario in limited localised injuries, such as sports, playground and low-altitude falls, which may be more common than cases with multiple abdominal wounds [55, 57]. In these cases CEUS can be of great value, revealing abdominal solid organ lesions not visible on baseline US and reaching high levels of sensitivity, specificity, and positive and negative predictive values [57]. Postcontrast injection, traumatic lacerations, and haematomas appear as nonenhancing areas [58, 59] (Figure 12). A limitation of CEUS for kidney trauma imaging is that it cannot rule out pelvicalyceal and ureter injuries, since contrast agents are not concentrated in the collecting system.

Although CEUS should be primarily used for unilateral limited injuries, multiple solid organ trauma can also be assessed [59]. In stable patients with a specific injury detected in the first 2-3 min, the remaining 2–5 min can be spent in scanning additional organs, dividing a full dose into 2-3 smaller doses. In trauma on the right side, with the patient in the left decubitus position, the right kidney can be assessed in the first 2 min and the liver in the remaining 3 min. In trauma on the left side, with the patient in the right decubitus position, the left kidney can be studied in the first 2 min and the spleen in the remaining 5 min (the spleen retains the contrast agent for as long as 7 min). CEUS cannot completely replace CT, but it may reduce its use as a screening method [60]. Stable, low-energy trauma patients with unilateral pain can be initially subjected to CEUS, possibly avoiding an emergency CT scan. Severe trauma cases should not be scanned with CEUS, but imaged with CECT if haemodynamically stable, or sent immediately to surgery if fluid is found on FAST examination and the patient is unstable. Finally, patients subjected to an initial CT and treated conservatively can be followed with CEUS with no additional CT performed [59].

### 6.7. Renal Artery Stenosis

Doppler examination of the renal arteries is still in many institutions the first imaging examination to be performed for assessing renal artery stenosis. There are published studies advocating the injection of US contrast agents in order to improve sensitivity of conventional Colour Doppler examination for the identification of the main renal arteries, with a 10% improvement [61] for correct location of the sample volume for detecting Doppler spectral tracings [15]. However, it is debatable if this slight amelioration is worth the extra time and cost, since patients may eventually be referred to CT/MR angiography of the renal arteries. For this reason, it may be concluded that routine use of CEUS offers no significant advantage for the evaluation of renal artery stenosis [15, 61].

### 6.8. Assessment of Percutaneous Ablation Therapy

CEUS, by characterising the microvasculature with perfusion analysis during the course of interventions, provides a lot of possibilities for modified therapeutic strategies. Percutaneous ablation is increasingly being used effectively for the management of patients with kidney tumours. These cases are often imaged with CECT and/or CEMR both for pretreatment evaluation in specific time points during followup after therapy. Although baseline, nonenhanced US may be useful for guiding the ablation procedure, it is not as effective for the assessment of ablation results [15]. Studies have shown that CEUS improves imaging of patients referred for renal tumour ablation [62], with similar accuracy to that of CT/MR for confirmation of treatment results [63, 64]. It offers detailed important information on tumour vascularity, thus improving orientation and guiding of the ablation needle. Furthermore, CEUS ameliorates evaluation of treatment therapeutic results [63, 64]. A delay of 5–10 min after the ablation is concluded allows the heat-generated gas and related artefacts to dissolve. Areas still showing contrast enhancement after ablation are considered as residual tumour. The examiner should be cautious not to misinterpret larger blood vessels surrounding the ablated region as a residual lesion [15]. For this reason, imaging results after therapy should be compared to pretreatment studies. Residual tumour is shown as a nodular or crescent-like area with contrast uptake, with close resemblance to pretreatment imaging findings [63].

Table 1 summarises the different indications for the use of CEUS in the kidneys, with findings on baseline US and postcontrast injection, along with corresponding literature references.

## 7. Limitations

The limitations of CEUS in the kidneys can be categorised into 3 groups.

The first group includes the known deficiencies of ultrasonography as a modality due to lesion location (obese patients, bowel gas interposition, etc.) that contrast agents cannot overcome. If a lesion is not seen on baseline examination, it will not be detected after post contrast injection either. Secondly, limitations exist for CEUS as a practice worldwide. Most US machines are not capable of imaging this technique [65], which is not included in structured Radiology training. Additional time is needed in order to place an intravenous catheter, while the drug's added cost should also be considered. Although, as already mentioned, patients with serious cardiopulmonary disease should not be scanned with CEUS [13, 66, 67], these cases are smaller in number in comparison to those with contraindication for contrast enhanced CT or MR because of anaphylactic history or renal failure. Finally, limitations exist for scanning the kidneys in particular: US contrast agents are not excreted to the pelvicalyceal system, while it is impossible to image the enhancement of both kidneys simultaneously, as in CT, MR or intravenous urography.

Despite these limitations, however, CEUS is used extensively worldwide for imaging a variety of renal pathologic entities with excellent results.

## Figures and Tables

**Table 1:** An overview of the pathological entities addressed in the paper with corresponding imaging details on baseline US and CEUS, as well as the references.

Pathological entities	Baseline US findings	CEUS findings	References
Differential diagnosis between solid renal masses and pseudotumours	Normal variants cannot always be differentiated from tumours	Tumour vascularity is different from normal parenchyma, at least in one vascular phase Any area enhancing differently is suspicious (Figure 1)	[15]
		Pseudotumours enhance parallel to the kidney parenchyma in all phases (Figure 2)	[14, 16]
	Solid tumours cannot be characterised as benign or malignant	Solid tumours do not show specific perfusion patterns to differentiate between benign and malignant lesions	[15]
	Colour Doppler has limitations in imaging neoplastic invasion of the renal vein and collecting system	Malignant renal vein thrombus enhances, while bland thrombus does not show contrast uptake. Enhancing material in the collecting system is characterised as neoplastic tissue contrary to nonenhancing infectious material (Figure 3)	[15, 19]

Differentiation between cystic and solid lesions	Colour Doppler has limitations in imaging perfusion in echogenic content of cysts	Solid hypovascular tumours enhance, even minimally, while debris does not (Figure 4)	[15, 17, 20]
		CEUS is superior to CT and MR for diagnosing cystic renal cell carcinoma	[8, 21, 22]

Characterisation of complex cystic renal masses	Colour Doppler has limitations in imaging perfusion in septa and nodules of cysts	CEUS shows enhancement in solid septa and nodules, with equal or superior diagnostic accuracy compared to CT for cyst classification using the Bosniak system (Figures 5–8)	[23, 36–38]
		CEUS is an alternative to CT for complex cysts followup	[20, 38, 42]

Renal ischaemia	Colour Doppler has limitations in imaging perfusion in small blood vessels with slow flow	CEUS is comparable to CECT for detecting parenchymal ischaemia. Infarcts appear as triangular or wedge-shaped areas with no contrast uptake (Figure 9)	[15, 16, 46]
		CEUS differentiates infarcts from parenchymal areas with diminished perfusion	[15]

Renal infections	B-mode US is needed to rule out the presence of calculi and urinary tract obstruction	Focal pyelonephritis shows areas of reduced enhancement. An abscess appears as a non-enhancing area with peripheral uptake (Figure 10)	[15]
		Puss in the collecting system or bladder shows no uptake (Figure 11)	[14]

Renal trauma	Baseline US is adequate for fluid detection but has low sensitivity for imaging traumatic lesions, which may be isoechoic and can be missed	CEUS reveals injuries not visible on baseline US as nonenhancing areas (Figure 12)	[51, 52, 57–59]
		Patients initially imaged with CT can be followed with CEUS	[59]

Renal artery stenosis	Doppler examination of renal arteries is the first imaging examination to be performed for assessing stenosis	Routine use of CEUS offers no significant advantage for renal artery stenosis evaluation	[15, 61]

Percutaneous ablation therapy assessment	Baseline US does not offer significant information	CEUS confirms treatment results, imaging remaining tumour vascularity. Areas still enhancing afterablation are considered as residual tumour	[62–64]
